# Prevalence of adenoid hypertrophy among 12-year-old children and its association with craniofacial characteristics: a cross-sectional study

**DOI:** 10.1186/s40510-023-00481-4

**Published:** 2023-09-11

**Authors:** Kwan Lok Tse, Fabio Savoldi, Kar Yan Li, Colman P. McGrath, Yanqi Yang, Min Gu

**Affiliations:** 1 Orthodontics, Division of Paediatric Dentistry and Orthodontics, Faculty of Dentistry, The University of Hong Kong, 2/F, Prince Philip Dental Hospital, 34 Hospital Road, Sai Ying Pun, Hong Kong SAR People’s Republic of China; 2 Clinical Research Centre, Faculty of Dentistry, The University of Hong Kong, 5/F, Prince Philip Dental Hospital, 34 Hospital Road, Sai Ying Pun, Hong Kong SAR People’s Republic of China; 3 Dental Public Health, Division of Applied Oral Sciences and Community Dental Care, Faculty of Dentistry, The University of Hong Kong, 1/F, Prince Philip Dental Hospital, 34 Hospital Road, Sai Ying Pun, Hong Kong SAR People’s Republic of China

**Keywords:** Adenoids, Lateral cephalometry, Upper airway, Children

## Abstract

**Background:**

Identifying the prevalence of adenoid hypertrophy (AH) and craniofacial factors associated with this condition requires studies with random sampling from the general population, and multiple criteria can be used for assessing AH on lateral cephalometric radiograph (LCR). The present analysis represents the first report performed according to these requirements in a large cross-sectional sample of children.

**Methods:**

LCRs of 517 12-year-old children (286 males, 231 females) randomly selected from the general population were retrospectively retrieved. AH was defined using three criteria (At/Nd, Ad-Ba/PNS-Ba, 1-Npaa/Npa), and twelve craniofacial variables were measured (SNA, SNB, ANB, Wits, Cd-Gn, MnP^SN, MxP^MnP, TPFH/TAFH, OPT^SN, C2ps-C4pi^SN, H-CV, H-FH). Skeletal characteristics were compared between children with and without AH using Mann–Whitney *U* test. Binary logistic regression (adjusted for sex and skeletal growth) was used to independently quantify the association between craniofacial factors and AH.

**Results:**

The prevalence of children with AH was 17.6% (according to At/Nd), 19.0% (according to Ad-Ba/PNS-Ba), and 13.9% (according to 1-Npaa/Npa). Children with AH presented greater antero-posterior jaw discrepancy (larger ANB, smaller SNB), greater mandibular divergence (larger MnP^SN), forward head posture (larger OPT^SN and C2ps-C4pi^SN), and anteriorly positioned hyoid bone (larger H-CV). Larger SNA (OR = 1.39–1.48), while smaller SNB (OR = 0.77–0.88) and Wits (OR = 0.85–0.87), were associated with greater likelihood of having AH, independently from the assessment method used.

**Conclusions:**

The prevalence of children with AH ranged from 13.9 to 19.0% based on LCR. Greater antero-posterior maxillo–mandibular discrepancy and mandibular retrusion were independently associated with higher likelihood of having AH.

**Supplementary Information:**

The online version contains supplementary material available at 10.1186/s40510-023-00481-4.

## Background

Adenoids are part of the lymphatic Waldeyer’s ring that is located in the superior nasopharynx. They grow after birth until the age of 5–7 years and shrink progressively thereafter [1, 2]. Adenoid hypertrophy (AH) can be physiological, secondary to infections, or a reaction to allergens [3] and, with a prevalence of about 34%, it is a common cause of upper airway obstruction in children [4]. AH is associated with increased nasal resistance to airflow and may lead to mouth-breathing [5], which may negatively impact the quality of life of children [6].

While nasopharyngeal endoscopy is the gold standard for diagnosing AH [7], lateral cephalometric radiograph (LCR) is a useful alternative tool to identify this condition because of its good diagnostic accuracy [7] and reliability [8]. Among numerous methods for assessing AH on LCR, the adenoid/nasopharyngeal ratio (At/Nd) [9], adenoid/retropalatal ratio (Ad-Ba/PNS-Ba) [10], and adenoid/nasopharyngeal area ratio (1-Npaa/Npa) [2] are based on relative measurements that account for individual size variations, leading to improved validity [11]. However, there is no consensus about which should be preferred, as authors reported inconsistent findings regarding the usefulness of both linear [12, 13] and area measurements [12, 14], and clinicians should consider the combination of multiple methods for assessing AH on LCR [15].

Since LCR also allows to assess craniofacial structures [16], it has been previously used for investigating the association between skeletal morphology and AH. However, conflicting results have been reported when children with AH were compared to controls [17, 18]. In fact, previous studies involved heterogeneous age groups that did not account for physiological changes in adenoids size [17, 18], and children were sampled from hospital departments, making them not representative of the general population [17, 18]. Overall, investigating the prevalence of AH and its associations with craniofacial structures requires studies on large samples selected via stratified random sampling from the general population. In addition, a cross-sectional design can reduce the bias related to physiological lymphoid tissue shrinkage [1, 2] and skeletal growth [19]. Twelve-year-old children represent a meaningful target population, as such age corresponds to an appropriate timing for the assessment of orthodontic treatment needs. Especially, given that AH may predispose children to sleep-disordered breathing [1], screening for this condition is advisable during treatment planning [20].

The objective of the present study was to estimate the prevalence of children with AH based on LCR, and to investigate the association between AH and craniofacial characteristics among 12-year-old children. It was hypothesised that children with increased antero-posterior jaw discrepancy, increased vertical facial height, forward craniocervical posture, and forward hyoid bone position had greater likelihood of having AH.

## Methods

### Participants

LCRs of 517 children were retrieved from a previous cross-sectional study that performed stratified sampling on 11 randomly selected schools in Hong Kong in the 1980s [21] and referred participants to Prince Philip Dental Hospital (Hong Kong SAR) for taking the X-ray. 12-year-old children, who had not received orthodontic treatment, and whose LCR was taken with similar methods were included. The study was approved by the Institutional Review Board of The University of Hong Kong / Hospital Authority Hong Kong West Cluster (UW12-405).

### Lateral cephalograms acquisition and analysis

**Fig. 1:**
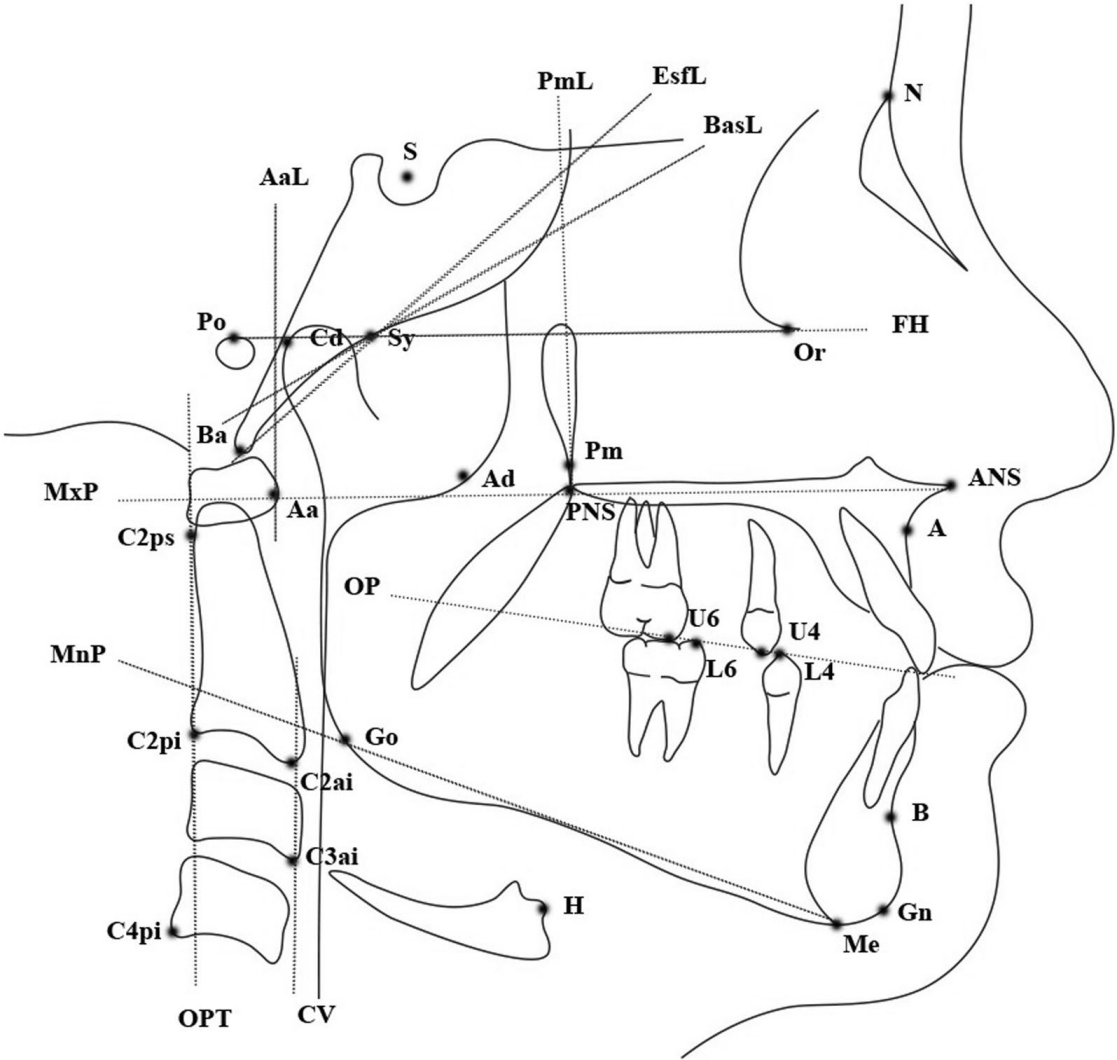
Cephalometric landmarks and lines

**Fig. 2:**
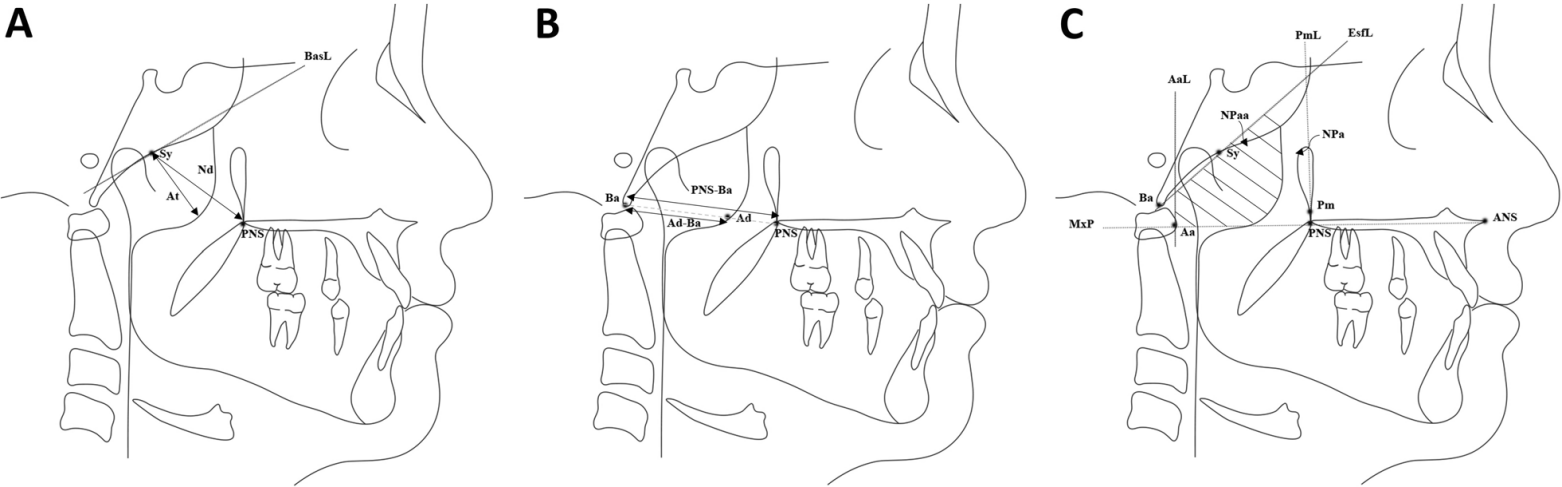
Cephalometric landmarks and measurements used to identify AH according to the three methods: At/Nd (**A**), Ad-Ba/PNS-Ba (**B**), and 1-Npaa/Npa (**C**)

One X-ray machine (GE1000, General Electric, Milwaukee, WI, USA) was used to obtain all LCRs, which were acquired in natural head posture [21]. Cephalometric analysis was carried out with computer software (CASSOS, SoftEnable Technology, Hong Kong SAR), while the nasopharyngeal areas were measured with graphical software (ImageJ) [22]. Linear and area measurements were adjusted according to a magnification of 8.75% and 18.27%, respectively. Cephalometric points and lines were identified (Fig. 1 and Table 1), and variables were measured (Table 2). AH was defined according to three parameters: the adenoid/nasopharyngeal ratio (At/Nd) [9], adenoid/retropalatal ratio (Ad-Ba/PNS-Ba) [10], and adenoid/nasopharyngeal area ratio (1-Npaa/Npa) [2] (Fig. 2). Children were classified as either having AH or not having AH according to cut-off values of 0.62 for At/Nd (> 0.62 indicating AH) [9], 0.60 for Ad-Ba/PNS-Ba (> 0.60 indicating AH) [10], and 0.35 for 1-Npaa/Npa (< 0.35 indicating AH) [2]. Skeletal maturity was assessed using the cervical vertebral maturation (CVM) method and growth stages were defined as pre-pubertal (CS1-CS2), pubertal (CS3-CS4), and post-pubertal (CS5-CS6) [19].

**Table 1:** Cephalometric landmarks and lines used to identify craniofacial structures and adenoids

Abbreviation	Name	Description
*Cephalometric points*		
A	A-point	The deepest point of the concavity of the anterior maxilla between ANS and the alveolar crest
Aa	Anterior atlas	The most anterior point of the atlas
Ad	Adenoids	The intersection between PNS-Ba and the anterior border of the adenoids
H	Hyoid	The most anterior–superior point on the body of the hyoid bone
ANS	Anterior nasal spine	The anterior tip of the bony apex of the maxillary complex
B	B-point	The deepest point in the concavity of the anterior mandible between the alveolar crest and the bony chin
Ba	Basion	The most inferior point of the anterior border of the foramen magnum
C2ai	2nd cervical vertebra A	The most anterior–inferior point of the second cervical vertebra
C2pi	2nd cervical vertebra B	The most posterior–inferior point of the second cervical vertebra
C2ps	2nd cervical vertebra C	The most posterior–superior point of the second cervical vertebra
C3ai	3rd cervical vertebra	The most anterior–inferior point of the third cervical vertebra
C4pi	4th cervical vertebra	The most posterior–inferior point of the fourth cervical vertebra
Cd	Condylion	The most posterior–superior point on the mandibular condyle
Gn	Gnathion	The most anterior–inferior point on the bony chin
Go	Gonion	The intersection of the tangents to the inferior and posterior borders of the mandible
U4	Maxillary first premolar cusp	The cusp tip of the maxillary first premolar
L4	Mandibular first premolar cusp	The cusp tip of the mandibular first premolar
U6	Maxillary first molar cusp	The mesial cusp tip of the maxillary first permanent molar
L6	Mandibular first molar cusp	The mesial cusp tip of the mandibular first permanent molar
Me	Menton	The most inferior point on the body chin
N	Nasion	The most anterior point of the fronto-nasal suture
Sy	Spheno-occipital synchondrosis	The anterior border of the spheno-occipital synchondrosis
Or	Orbitale	The most inferior point of the inferior borders of the bony orbit
Pm	Pterygo-maxillare	The most inferior point of the pterygomaxillary fissure
PNS	Posterior nasal spine	The posterior tip of the bony apex of the maxillary complex
Po	Porion	The most superior point of the external auditory meatus
S	Sella	The geometric centre of the sella turcica
*Cephalometric lines*		
CV	Cervical vertebrae	The line joining C2ai and C3ai
AaL	Anterior atlas line	The line perpendicular to MxP and passing through Aa
EsfL	Sphenoid line	The line tangent to the inferior border of the sphenoid bone and passing through Ba
FH	Frankfort horizontal plane	The line joining Or and Po
MnP	Mandibular plane	The line joining Me and Go
MxP	Maxillary plane	The line joining ANS and PNS
OP	Occlusal plane	The line joining the midpoint between U4 and L4 with the midpoint between U6 and L6
OPT	Odontoid process tangent	The line joining C2ps and C2pi
PmL	Pterygomaxillary line	The line perpendicular to MxP and passing through Pm
BasL	Basioccipital line	The line representing the anterior margin of the basioccipital bone

All bilateral points were marked by choosing the average between the left and right side

**Table 2:** Cephalometric variables used for craniofacial structures and adenoids

Abbreviation		Name	Description
*Measured variables (for calculation of variables included in the analysis)*			
At	mm	Nasopharyngeal soft tissue thicknesses of adenoid	The largest distance between BasL and the anterior border of the adenoids
Ad-Ba	mm	Retropalatal soft tissue thickness of adenoid	The distance between Ad and Ba
Nd	mm	Nasopharyngeal depth	The distance between PNS and Sy
Npa	mm^2^	Nasopharyngeal area	The area bounded by PmL, EsfL, MxPl and AaL
Npaa	mm^2^	Nasopharyngeal adenoid area	The area occupied by the adenoids within Npa
PNS-Ad	mm	Airway sagittal depth	The distance between PNS and Ad
TAFH	mm	Total anterior facial height	The distance between N and Me
TPFH	mm	Total posterior facial height	The distance between S and Go
*Measured variables (directly included in the analysis)*			
ANB	°	ANB angle	The angle between N-A line and N-B line
C2ps-C4pi^SN	°	Total craniocervical angle	The angle between C2ps-C4pi line and S–N line
Cd-Gn	mm	Mandibular length	The distance between Cd and Gn
H-CV	mm	Horizontal position of hyoid bone	The length of the segment from H to CV on a line that is parallel to FH
H-FH	mm	Vertical position of hyoid bone	The distance between H and FH
MnP^SN	°	Mandibular plane angle	The angle between MnP and S–N line
MxP^MnP	°	Maxillo-mandibular planes angle	The angle between MxP and MnP
OPT^SN	°	Upper craniocervical angle	The angle between OPT and S-N line
SNA	°	SNA angle	The angle between S-N line and N-A line
SNB	°	SNB angle	The angle between S-N line and N-B line
Wits	mm	Wits appraisal	The distance between the projections of A and B on OP
*Calculated variables (included in the analysis)*			
At/Nd	%	Adenoid/nasopharyngeal ratio	The ratio of At to Nd
Ad-Ba/PNS-Ba	%	Adenoid/retropalatal ratio	The ratio of Ad-Ba to PNS-Ba
1-Npaa/Npa	%	Adenoid/nasopharyngeal area ratio	1 minus the ratio of Npaa to Npa
TPFH/TAFH	%	Jarabak ratio	The ratio of TPFH to TAFH

### Calibration and method error

The primary assessor (KLT) carried out all measurements after calibration with a secondary assessor (GM). The calibration consisted of tracing and discussing 10 LCR and repeating the procedure until an intraclass correlation coefficient (ICC) > 0.75 was achieved. For intra-assessor reliability, 20% of the LCRs (*n* = 104) were re-measured by the primary assessor after a wash-out period of about 2 weeks.

### Sample size calculation

The sample size was calculated based on a binary logistic regression model with the presence of AH as dependent variable and 14 independent variables (2 covariates to adjust the model for confounding effects, and 12 factors to investigate their effect on the dependent variable). According to a reported prevalence of AH of 31.76% in children and adolescents [17], and requiring at least 10 subjects for each independent variable [23], the sample size was calculated as *N* = (14 × 10)/0.3176 = 441. Given the retrospective nature of the study, all the 517 records fulfilling the inclusion criteria were included.

### Data analysis

The measurement error was calculated with Dahlberg’s formula [24]. ICC was used to calculate the intra-assessor agreement of continuous variables (< 0.50 “poor”, 0.50–0.74 “moderate”, 0.75–0.90 “good”, > 0.90 “excellent” [25]). Cohen’s kappa (*K*) was used to calculate the intra-assessor agreement of growth stages and the agreement among the three assessment methods of AH (≤ 0.20 “poor”, 0.21–0.40 “slight”, 0.41–0.60 “fair”, 0.61–0.80 “good”, 0.81–0.92 “very good”, ≥ 0.93 “excellent” [26]). Chi-square test was used to determine variations in the distribution of children by sex and growth stages with respect to the presence of AH, independently for each of the three diagnostic methods. Normal distribution of continuous data was assessed using Shapiro–Wilk test, and Mann–Whitney *U* test was used to compare the craniofacial variables between children with and without AH, independently for each of the three diagnostic methods. Binary logistic regression (with covariates adjustment for growth stage and sex) was used to assess the independent contribution of each craniofacial factor to the presence of AH. Three binary logistic regression models, one for each diagnostic method, were developed. Statistical analysis was performed using SPSS 27.0 (IBM, New York, USA), at significance *α* = 0.05.

## Results

### Sample characteristics

A total of 517 children, 286 males (55.3%) and 231 females (44.7%), were included. Regarding skeletal growth, 121 males (92.4%) and 10 females (7.6%) were in pre-pubertal stage, 157 males (55.7%) and 125 females (44.3%) were in pubertal stage, while 8 males (7.7%) and 96 females (92.3%) were in post-pubertal stage.

### Measurements error and agreement

The measurement error was < 1.0° for angular measurements, < 1.0 mm for linear measurements, and < 2.0% for ratios. Regarding the independent variables, the intra-assessor agreement was very good for skeletal growth (*K* = 0.823), and very good to excellent for craniofacial structures (ICC = 0.908–0.990). Regarding the methods used for identifying children with AH, the intra-assessor agreement of each method was excellent (ICC = 0.956–0.999), and the agreement among the methods was very good between At/Nd and Ad-Ba/PNS-Ba (*K* = 0.877), very good between At/Nd and 1-Npaa/Npa (*K* = 0.818), and good between Ad-Ba/PNS-Ba and 1-Npaa/Npa (*K* = 0.790) (Appendix Tables 6, 7).

### Prevalence of adenoid hypertrophy

Ninety-one children (17.6%, 49 males and 42 females) were classified as having AH according to At/Nd, 98 (19.0%, 52 males and 46 females) according to Ad-Ba/PNS-Ba, and 72 (13.9%, 35 males and 37 females) based on 1-Npaa/Npa. There was no difference in sex distribution or skeletal growth stage between children with and without AH, independently from the assessment method used (Table 3).

**Table 3:** Distribution of sex and growth stages based on adenoid hypertrophy identified with three different methods

	At/Nd					Ad-Ba/PNS-Ba					1-Npaa/Npa				
	Non-hypertrophic		Hypertrophic			Non-hypertrophic		Hypertrophic			Non-hypertrophic		Hypertrophic		
n	426		91			419		98			445		72		
%	82.4		17.6			81.0		19.0			86.1		13.9		
	n	%	n	%	*p* value	n	%	n	%	*p* value	n	%	n	%	*p* value
*Sex*															
Male	237	55.6	49	53.8	0.816	234	55.8	52	53.1	0.652	251	56.4	35	48.6	0.250
Female	189	44.4	42	46.2		185	44.2	46	46.9		194	43.6	37	51.4	
*Growth stage*															
Pre-pubertal	108	25.4	23	25.3	0.995	108	25.8	23	23.5	0.840	115	25.8	16	22.2	0.806
Pubertal	232	54.5	50	54.9		226	53.9	56	57.1		241	54.2	41	56.9	
Post-pubertal	86	20.2	18	19.8		85	20.3	19	19.4		89	20.0	15	20.8	

Chi-square test was used to compare either sex or growth stage distribution within non-hypertrophic and hypertrophic patients

### Association between adenoid hypertrophy and craniofacial characteristics

Regarding horizontal measurements, ANB of children with AH was larger (for all methods) and SNB was smaller (for At/Nd and 1-Npaa/Npa). The logistic regression showed that ANB, SNB, and Wits were significant predictors in all models (for each degree of increase of ANB, children were 38.8–47.5% more likely of having AH; for each degree of decrease in SNB, they were 13.6–29.3% more likely to have AH; and for each millimetre of decrease in Wits, they were 15.6–18.0% more likely of having AH). Larger Cd-Gn was also significant (for At/Nd and 1-Npaa/Npa). For vertical measurements, MnP^SN was larger in children with AH (for At/Nd). However, no vertical parameter was significant in the logistic regression. Regarding craniocervical posture, both OPT^SN (for At/Nd) and C2ps-C4pi^SN (for At/Nd and Ad-Ba/PNS-Ba) were larger in children with AH. However, no craniocervical parameter was significant in the logistic regression. For the hyoid bone position, H-CV was larger among children with AH (for At/Nd). The logistic regression showed that children with more anteriorly positioned hyoid bone were more likely of having AH (for At/Nd) (Tables 4, 5).

**Table 4:** Comparison of craniofacial characteristics between children with and without adenoid hypertrophy, for each of the three assessment methods

Variable	Unit	At/Nd								Ad-Ba/PNS-Ba								1-Npaa/Npa							
		Non-hypertrophic			Hypertrophic			Δ		Non-hypertrophic			Hypertrophic			Δ		Non-hypertrophic			Hypertrophic			Δ	
		*n* = 426			*n* = 91					*n* = 419			*n* = 98					*n* = 445			*n* = 72				
		Median	LQ	UQ	Median	LQ	UQ	Median	*p* value	Median	LQ	UQ	Median	LQ	UQ	Median	*p* value	Median	LQ	UQ	Median	LQ	UQ	Median	*p* value
*Horizontal*																									
SNA	°	85.5	82.6	88.3	86.0	82.8	88.2	0.5	0.959	85.5	82.5	88.1	86.1	82.9	88.6	0.5	0.336	85.5	82.8	88.3	85.1	82.6	88.1	− 0.4	0.506
SNB	°	81.3	78.9	83.9	80.1	77.2	82.5	− 1.2	**0.005**	81.2	78.8	83.7	80.4	77.4	83.6	− 0.9	0.132	81.2	78.8	83.9	79.9	77.3	82.5	− 1.4	**0.008**
ANB	°	4.2	2.9	5.6	5.8	3.7	7.1	1.6	**< 0.001**	4.2	2.9	5.6	5.8	3.6	6.7	1.6	**< 0.001**	4.2	2.9	5.7	5.8	3.7	6.6	1.6	**0.001**
Wits	mm	− 3.8	− 6.4	− 1.1	− 3.1	− 6.2	− 0.6	0.7	0.337	− 3.8	− 6.3	− 1.0	− 3.4	− 7.0	− 0.7	0.5	0.774	− 3.8	− 6.4	− 1.0	− 3.4	− 6.2	− 0.8	0.5	0.624
Cd-Gn	mm	110.6	107.2	115.1	112.0	108.8	115.3	1.5	0.104	110.7	107.2	115.1	111.6	108.3	115.3	0.9	0.176	110.7	107.2	115.1	111.6	108.7	115.3	0.9	0.201
*Vertical*																									
MnP^SN	°	33.8	30.3	36.5	35.0	30.4	39.5	1.2	**0.030**	33.9	30.3	36.6	34.3	30.4	39.2	0.4	0.131	33.9	30.2	36.8	34.8	30.8	39.1	0.9	0.083
MxP^MnP	°	26.5	23.4	29.1	27.8	23.6	31.2	1.3	0.052	26.5	23.3	29.1	27.6	23.8	31.1	1.1	0.054	26.6	23.3	29.3	27.9	24.0	31.1	1.3	0.070
TPFH/TAFH	%	0.7	0.7	0.7	0.7	0.6	0.7	0.0	0.097	0.7	0.7	0.7	0.7	0.6	0.7	0.0	0.191	0.7	0.7	0.7	0.7	0.6	0.7	0.0	0.124
*Craniocervical*																									
OPT^SN	°	97.1	91.6	101.8	99.4	93.0	105.1	2.4	**0.014**	97.2	91.7	101.9	98.7	93.0	105.1	1.5	0.084	97.2	91.8	102.1	99.2	93.0	105.1	2.0	0.097
C2ps-C4pi^SN	°	99.5	94.5	104.2	102.1	95.4	108.5	2.6	**0.008**	99.5	94.5	104.2	101.4	94.7	107.5	1.8	**0.042**	99.7	94.5	104.2	101.9	94.8	107.7	2.1	0.052
*Hyoid bone*																									
H-CV	mm	32.4	30.1	35.3	33.9	30.4	36.0	1.5	**0.022**	32.4	30.1	35.3	33.6	30.4	36.1	1.2	0.058	32.4	30.3	35.3	33.2	30.2	35.9	0.8	0.234
H-FH	mm	81.8	76.6	85.9	82.8	77.2	89.5	1.0	0.072	81.9	76.8	86.0	82.1	76.8	89.3	0.2	0.255	81.9	76.9	86.2	81.7	75.3	89.5	− 0.3	0.577

LQ lower quartile (25th percentile), UQ upper quartile (75th percentile)

Significant *p* values are reported in bold

Mann–Whitney *U* test was used to compare non-hypertrophic and hypertrophic patients

Δ = difference between hypertrophic and non-hypertrophic patients

**Table 5:** Binary logistic regression models showing the relationship between the dependent factors and adenoid hypertrophy, for each of the three assessment methods

		At/Nd			Ad-Ba/PNS-Ba			1-Npaa/Npa		
		AOR	*p* value	Pseudo *R*^2^	AOR	*p* value	Pseudo *R*^2^	AOR	*p* value	Pseudo *R*^2^
*Covariates adjustments*										
				0.148			0.103			0.121
Sex	Male	1.000	0.303		1.000	0.445		1.000	0.071	
	Female	1.397			1.263			1.871		
Growth stage	Pre-pubertal	1.000	0.921		1.000	0.727		1.000	0.788	
	Pubertal	1.002			0.906			1.074		
	Postpubertal	1.108			1.139			1.247		
*Craniofacial factors*										
SNB		0.818	**0.003**		0.880	**0.044**		0.773	**< 0.001**	
ANB		1.475	**< 0.001**		1.404	**< 0.001**		1.388	**< 0.001**	
Wits		0.853	**0.003**		0.865	**0.005**		0.847	**0.005**	
Cd-Gn		1.075	**0.014**		1.052	0.064		1.083	**0.011**	
MnP^SN		0.943	0.532		0.897	0.226		0.869	0.171	
MxP^MnP		1.025	0.729		1.083	0.249		1.124	0.142	
TPFH/TAFH		7.119	0.779		0.372	0.882		12.907	0.737	
OPT^SN		0.947	0.312		0.927	0.141		0.917	0.137	
C2ps-C4pi^SN		1.064	0.259		1.093	0.089		1.083	0.179	
H-CV		1.073	**0.042**		1.058	0.094		1.064	0.093	
H-FH		0.980	0.349		0.972	0.183		0.965	0.139	

AOR adjusted odd ratio, *R*^*2*^ coefficient of determination

Significant *p* values are reported in bold

SNA was removed from the model because of redundancy with SNB and ANB (and it showed no statistical significance in the group comparison)

## Discussion

Adenoids have been assessed on LCR since 1946 [27], and numerous parameters have been proposed to identify children with AH. The present study adopted three methods, with the first based on the ratio between the maximum thicknesses of the adenoids (measured from the basilar part of the occipital bone) and the width of the nasopharynx (measured along the line connecting the posterior nasal spine with the spheno-occipital synchondrosis). This parameter, named At/Nd, was first described by Fujioka et al*.* [9]. An advantage of using ratio measurements is that the adenoidal size is considered with respect to the individual nasopharyngeal capacity. In children, At/Nd has excellent specificity (95%) and positive predictive value (94%), but low sensitivity (41%) and negative predictive value (39%) [28]. At/Nd has also been shown to correlate well with clinical symptoms and weight of surgically removed adenoids [29]. The second parameter consisted of the ratio between the thickness of the adenoids and the width of the nasopharynx (both measured along the line connecting the posterior nasal spine with Basion). This ratio, named Ad-Ba/PNS-Ba and proposed by Kemaloglu et al*.* [10], is similar to At/Nd but measured along a different line that is more representative of the retropalatal area. Among children, it has excellent specificity (97%) and positive predictive value (97%), but moderate sensitivity (71%) and negative predictive value (70%) [11]. The third parameter represented the proportion of the nasopharyngeal area that is not occupied by the adenoids (1-Npaa/Npa), as proposed by Handelman and Osborne [2]. It has excellent specificity (94%) and positive predictive value (95%), but moderate sensitivity (75%) and negative predictive value (72%) [11]. In general, the present study showed a prevalence of children with AH between 13.9% and 19.0%, with good agreement among the three measurements methods. Of note, the calculated prevalence was lower than the 34.46% reported by a previous meta-analysis of studies using nasoendoscopy in children [4]. The reason for this difference could be due to the lower sensitivity of LCR compared to nasoendoscopy, with a reported pooled sensitivity of 86% in children [7]. In particular, the only two identified studies using nasoendoscopy in randomly selected samples included children between 5- and 14-year-old [4], further justifying differences between previously reported values and the present cross-sectional study among 12-year-old children. No difference was present in the prevalence of AH between males and females, in agreement with a former investigation [30].

Hypertrophic adenoids may obstruct the nasopharynx, forcing the child to breathe through the mouth, which may lead to the characteristic “adenoid facies” [31]. However, the relationship between AH and craniofacial morphology should be considered with caution, as patients may not show the expected “mouth-breathing dental stereotype” [18], and it is time for scientific studies to critically analyse confounders and statistical findings. In fact, multiple genetic and epigenetic factors may affect craniofacial growth [32]. In this complex scenario, despite the comparison of the median values of a variable (e.g. SNA) between two groups (e.g. children with AH versus without AH) provides information about the presence of an association, it does not quantify its strength and it does not account for confounders [33]. The present study showed that increased maxillo-mandibular sagittal discrepancy with retruded and hyperdivergent mandible was associated with AH, which was in partial agreement with the findings of a previous study in children with sleep-disordered breathing [34]. In addition, in the present work, forward head posture and forward position of the hyoid bone were present in children with AH. Although these findings are in contrast to previous works that found no significant evidence of such associations [17, 35], the former studies involved small samples (i.e. small statistical power), participants were recruited from hospitals (i.e. not representative of the general population), and they involved patients of a wide age-range (i.e. confounding factor due to changes in adenoids size). This said, a simple group comparison does not allow proper investigation of the association between AH and craniofacial characteristics. While “risk” is the probability of occurrence of an event (e.g. the chance of having AH), “odds” is the ratio of the probability of occurrence of an event to the probability of that event not occurring (e.g. the chance of having AH with respect to the chance of not having it). The “odds ratio” (OR) is the ratio of odds of an event (e.g. odds of having AH) in one group (e.g. children with retruded mandible) versus the odds of that event in another group (e.g. children without retruded mandible) [36]. Furthermore, multiple logistic regression calculates the “adjusted OR”, which provides the independent effect of a variable (e.g. SNA) while holding all other variables fixed (e.g. SNB, Wits) on a binomial outcome (e.g. having versus not having AH) [37]. The present study showed increased adjusted OR of having AH among children with skeletal Class II tendency characterised by retruded mandible and decreased Wits appraisal. Despite a former study performing a regression analysis found no significant relationship between craniofacial proportions and adenoid size, of note no horizontal skeletal parameter was included [38].

Overall, LCR accompanied by medical history examination may be useful for the screening of patients with suspected AH, providing indications of the need for referral to otorhinolaryngologist [39]. Given the excellent specificity but low/moderate sensitivity of the proposed methods (i.e. non-negligible chance to miss a patient truly having AH), orthodontists may not rely on a single parameter for identifying children with AH, and a combined assessment using linear and area measurements may be advisable. Since the onset of AH in children is multifactorial, with asthma, allergic rhinitis, and atopic dermatitis among the risk factors [30], orthodontic options should be integrated in a multidisciplinary treatment planning [40]. Appropriate timing for orthodontic intervention, with respect to medical and/or surgical options, should be discussed with other specialists for optimising clinical outcomes while minimising the burden to patients (Additional file 1).

### Limitations

Although the use of a retrospective archive from the 1980s allowed for analyses that would be impossible today for ethical reasons, the prevalence of AH may have changed over the past forty years. A recent meta-analysis [4] showed that only one 2005 study from Brazil [41] and another 2008 study from Turkey [42] included random samples from the general population. Even broadening the inclusion criteria, no study on AH prevalence was performed in Hong Kong and high heterogeneity was found between studies [4]. Furthermore, the incidence of adenotonsillectomy may be determined by factors other than the prevalence of AH [43] and local policies may influence environmental factors [44], limiting the use of adenotonsillectomy as a proxy for AH. Therefore, currently available data may not allow speculating on possible trends of changes in prevalence of AH over time in Hong Kong.

Further longitudinal studies are necessary to investigate possible causal relationships and the direction of such cause-effects between the development of altered craniofacial features and the onset of AH (e.g. whether a genetically small nasal cavity may lead to mouth-breathing and frequent upper respiratory tract infections causing AH [45, 46], or whether AH may lead to mouth-breathing and altered craniofacial development [5, 31]).

## Conclusions

- The prevalence of AH among 12-year-old children ranged between 13.9 and 19.0%, based on the method used for its assessment on LCR.
- In this population, greater antero-posterior maxillo-mandibular discrepancy and mandibular retrusion were associated with higher likelihood of having AH, which should be considered during orthodontic treatment planning.
- LCR is commonly used in orthodontic patients and, despite being a static 2D assessment with biohazard related to ionising radiations, it may help in the identification of those with AH for further consideration with other specialists.

### Supplementary Information

**Additional file 1**. Complete raw dataset of the measurements used for the analysis presented in the study.

## Data Availability

All data generated or analysed during this study are included in this published article and its supplementary information files (Additional file 1: Raw dataset).

## Appendix

See Tables 6 and 7.

**Table 6:** Intra-examiner agreement estimated with intraclass correlation coefficient (ICC), and respective measurement errors estimated with Dahlberg’s formula

	Unit	ICC			Dahlberg’s error
		Value	95% CI	*p* value	Value
*Assessment methods (continuous)*					
At/Nd	%	0.992	0.988–0.995	< 0.001	0.86
Ad-Ba/PNS-Ba	%	0.956	0.936–0.970	< 0.001	1.53
1-Npaa/Npa	%	0.999	0.999–1.000	< 0.001	0.16
*Independent variables (continuous)*					
SNA	°	0.961	0.899–0.981	< 0.001	0.63
SNB	°	0.968	0.946–0.980	< 0.001	0.58
ANB	°	0.908	0.867–0.937	< 0.001	0.75
Wits	mm	0.945	0.898–0.968	< 0.001	0.94
Cd-Gn	mm	0.967	0.943–0.980	< 0.001	0.96
MnP^SN	°	0.982	0.967–0.990	< 0.001	0.69
MxP^MnP	°	0.962	0.943–0.975	< 0.001	0.89
TPFH/TAFH	%	0.950	0.927–0.966	< 0.001	0.86
OPT^SN	°	0.990	0.982–0.994	< 0.001	0.73
C2ps-C4pi^SN	°	0.990	0.983–0.993	< 0.001	0.74
H-CV	mm	0.913	0.873–0.940	< 0.001	0.95
H-FH	mm	0.982	0.974–0.988	< 0.001	0.91

ICC intraclass correlation coefficient, CI confidence interval

**Table 7:** Intra-examiner and inter-method agreement estimated with Cohen’s kappa (*K*)

	Unit	*K*		
		Value	SE	*p* value
*Agreement within assessment methods (binary)*				
At/Nd	0/1	0.886	0.064	< 0.001
Ad-Ba/PNS-Ba	0/1	0.892	0.061	< 0.001
1-Npaa/Npa	0/1	0.960	0.040	< 0.001
*Agreement between assessment methods (binary)*				
At/Nd versus Ad-Ba/PNS-Ba	0/1	0.877	0.028	< 0.001
At/Nd versus 1-Npaa/Npa	0/1	0.818	0.035	< 0.001
Ad-Ba/PNS-Ba versus 1-Npaa/Npa	0/1	0.790	0.037	< 0.001
*Independent variables (categorical)*				
Growth stage	1/2/3	0.823	0.049	< 0.001

*K* = Cohen's kappa, SE = standard error

## Abbreviations

1-Npaa/Npa: Adenoid/nasopharyngeal area ratio
2D: Two-dimensional
Ad-Ba/PNS-Ba: Adenoid/retropalatal ratio
AH: Adenoid hypertrophy
At/Nd: Adenoid/nasopharyngeal ratio
CVM: Cervical vertebral maturation
ICC: Intraclass correlation coefficient
K: Kohen’s Kappa coefficient
LCR: Lateral cephalometric radiograph
OR: Odds ratio
